# Aktuelle Entwicklungen in der Schlafforschung und Schlafmedizin – eine Einschätzung der AG „Pädiatrie“

**DOI:** 10.1007/s11818-022-00383-3

**Published:** 2022-08-16

**Authors:** Ekkehart Paditz, Alfred Wiater, Osman Ipsiroglu, Mirja Quante, Silvia Müller-Hagedorn, Bernhard Hoch, Thomas Erler, Julian Mollin, Barbara Schneider, Christian F. Poets

**Affiliations:** 1 Zentrum für Angewandte Prävention®, Blasewitzer Str. 41, 01307 Dresden, Deutschland; 2 Grosse Neugasse 6, 50667 Köln, Deutschland; 3 BC Children’s Hospital/BCCH Research Institute, 4500 Oak St, BC V6H 3N1 Vancouver, Kanada; 4 Universitätsklinik für Kinder- und Jugendheilkunde, Währinger Gürtel 18–20, 1090 Wien, Österreich; 5 Universitätsklinikum Tübingen, Calwerstr. 7, 72076 Tübingen, Deutschland; 6 Department für Zahn‑, Mund- und Kieferheilkunde, Klinik für Kieferorthopädie, Universitätsklinikum Freiburg, Hugstetterstr. 55, 79106 Freiburg, Deutschland; 7 Erlenweg 3, 86486 Bonstetten, Deutschland; 8 Klinikum Westbrandenburg, Standort Potsdam, Charlottenstr. 72, 14467 Potsdam, Deutschland; 9 Sozialpädiatrisches Zentrum Landshut am Kinderkrankenhaus St. Marien gGmbH, Grillparzerstr. 9, 84036 Landshut, Deutschland

## Kinder sind keine kleinen Erwachsenen

Zum Thema „Kind und Schlaf“ sind in den letzten Jahren durch die Kinderschlafmedizin und -psychologie, durch die Kinder- und Jugendpsychiatrie und seitens der Psychotherapeuten für Kinder und Jugendliche zahlreiche konkrete Fragen zur Diagnostik, Pathophysiologie, Chronobiologie, Epidemiologie, Therapie, Prävention, Gesundheitsökonomie und auch zur Traumforschung und Geschichte der Medizin bearbeitet und vertieft geklärt worden [1–8]. Lisa Mattriciani ist im Ergebnis eines breit angelegten Meta-Reviews jedoch zu dem Ergebnis gekommen, dass die Komplexität des Themas und die darin enthaltenen Interaktionen bisher noch nicht ausreichend untersucht worden sind [1]. Ganz in diesem Sinne erinnerte David Gozal kürzlich an den berühmten Arzt Moses Maimonides aus dem 12. Jahrhundert: „Der Arzt soll nicht die Krankheit behandeln, sondern den Patienten, der daran leidet.“ Gleichzeitig ermunterte Gozal mit Bezug auf den weiterhin offenen Wettbewerb zwischen der ambulanten und der stationären Polygraphie/Polysomnographie dazu, nicht stehen zu bleiben und geltende diagnostische und therapeutische Standards dem ständigen Wissenszuwachs anzugleichen: „adjust, adjust, adjust“ [9, 10].

Was gibt es Neues? Warum sind Kinder keine verkleinerten Erwachsenen? Wie belastbar ist die empirische Basis der freudschen Traumforschung? Darüber wird im folgenden Text kurz berichtet.

## Ergebnisse und Schwerpunkte der letzten 5 Jahre

Am 07.07.2022 ist die Neufassung der AWMF(Arbeitsgemeinschaft der Wissenschaftlichen Medizinischen Fachgesellschaften)-Leitlinie der Arbeitsgruppe Pädiatrie der DGSM (Deutsche Gesellschaft für Schlafforschung und Schlafmedizin) zum Thema „Prävention des plötzlichen Säuglingstodes“ im Konsens verabschiedet worden. Dank umfangreicher Informationskampagnen ist es gelungen, die Häufigkeit des plötzlichen Säuglingstodes (SIDS [sudden infant death syndrome]) in Deutschland um 93 % zu senken. In absoluten Zahlen bedeutet das: Im Jahr 1991 sind noch 1285 Säuglinge plötzlich und unerwartet gestorben, 2020 lag diese Zahl bei 84 (www.gbe-bund.de) [11]. Schon lange wird vermutet, dass Weckreaktionen und bestimmte Schutzreflexe wie Arousal und Gasping bei den betroffenen Säuglingen gestört sein könnten und/oder in Bauchlage nicht ausreichend wirksam werden können. Aus Australien wurde kürzlich auf der Grundlage einer Fall-Kontroll-Studie berichtet, dass vegetative Funktionen, die an die Verwertung von Acetylcholin gebunden sind, bei SIDS-Opfern gestört sein könnten [12]. Ob sich diese These in weiteren Studien bestätigen lässt, bleibt abzuwarten [13].

*Obstruktive Schlafapnoen* sind auch im Kindes- und Jugendalter die häufigste Ursache schlafbezogener Atmungsstörungen. In Tübingen ist eine individuell angepasste „Gaumenplatte mit Sporn“ entwickelt worden, die sich bei zahlreichen Kindern mit kraniofazialen Fehlbildungen bewährt hat und auf dem besten Wege ist, ein internationaler Behandlungsstandard zu werden [14, 15]. Hinweise zur Herstellungsmethode siehe: [16, 17] Kieferchirurgische Optionen, nasales CPAP, Intubation und Tracheotomie sind damit in vielen Fällen nicht mehr erforderlich. Es handelt sich nicht nur um eine symptomatische mechanische Weitung der engen und kollabierenden oberen Atemwege, sondern auch um einen kurativen Behandlungsansatz: Da sich die Schädelstrukturen noch im Wachstum befinden, werden skelettale Entwicklungsimpulse gesetzt, die zum Wachstum des Unterkiefers beitragen [18]. Die Tübinger Gaumenplatte wurde zunächst für Kinder mit Pierre-Robin-Sequenz (PRS) entwickelt, die durch Mikro- und Retrognathie, Glossoptose und obstruktive Atmungsstörung gekennzeichnet ist. Inzwischen wird diese Therapie auch bei Kindern mit PRS-assoziierten kraniofazialen Fehlbildungen erfolgreich eingesetzt.

Bei Kindern und Jugendlichen mit Down-Syndrom (Trisomie 21) treten obstruktive Schlafapnoen besonders häufig auf [19]. In einer retrospektiven Analyse von 223 Kindern mit Down-Syndrom, die wiederholt über mehrere Jahre im Kinderschlaflabor Tübingen untersucht worden waren, hatten 56 % der Patienten bei der Erstuntersuchung schlafbezogene Atmungsstörungen. Interessanterweise entwickelten 26 % der zunächst unauffälligen Kinder erst im Laufe der Folgeuntersuchungen eine obstruktive Schlafapnoe [20]. Die umfangreiche AWMF-Leitlinie „Down-Syndrom im Kindes- und Jugendalter“ [21] wird zurzeit im Konsens mit mehr als 30 Fachgesellschaften, Berufsverbänden und Selbsthilfegruppen aktualisiert. Die Kodierungshäufigkeit der ICD G47.3 (Schlafapnoe) bei Personen mit Trisomie 21 ist im stationären Bereich seit dem Erscheinen der ersten Auflage dieser Leitlinie (2016) signifikant angestiegen [8].

Das wichtige Thema des perioperativen Managements bei Kindern mit obstruktiver Schlafapnoe im Rahmen HNO-chirurgischer Maßnahmen wurde 2021 in einer S1-Leitlinie der AWMF ausführlich abgehandelt [22, 23].

*Handy, Gamen, TV und anderer Bildschirmkonsum* sind zu einem der Hauptthemen geworden, denen sich die pädiatrische Schlafmedizin und -psychologie zu stellen hat. Insomnien und die Disruption chronobiologischer Rhythmen sind ungewollte Nebenwirkungen der Digitalisierung [24, 25]. Hinzu kommt, dass Dialogstrukturen in Familien und Peergruppen labilisiert, gestört oder gar nicht erst entwickelt werden, was sich natürlich auch auf die Häufigkeit von Ein- und Durchschlafstörungen auswirkt. Erste Studien zeigen, dass hier auch Smartphone-App-basierte Interventionen funktionieren könnten [26]. Praxisorientierte, mehrdimensionale Behandlungsstrategien sind entwickelt und evaluiert worden, die zunehmend mehr in therapeutische Angebote implementiert werden [27].

Zahlreiche Studien zeigen, dass ein bedeutsamer Teil der* Konsolidierung von Gedächtnisinhalten im Schlaf* erfolgt [28, 29], wobei das sich entwickelnde kindliche Gehirn offensichtlich anderen biologisch verankerten Programmen folgt, als dies beim Erwachsenen der Fall ist. Diese Daten werden durch die Ergebnisse zum prä- und postnatalen Lernen ergänzt und zunehmend vertieft.

Zu beachten ist:* ADHS* ist nicht immer ADHS (Aufmerksamkeits- und Hyperaktivitätsstörung) [30, 31]. Die Bedeutung des Schlafes wird in der ADHS-Diagnostik oft nicht ausreichend berücksichtigt [32, 33]. Seitens der Arbeitsgruppe Pädiatrie der DGSM wird im Verbund mit den Österreichischen Gesellschaften für Schlafmedizin, Kinder- und Jugendheilkunde und Kinder- und Jugendpsychiatrie, Psychosomatik und Psychotherapie dringend darauf aufmerksam gemacht, dass man differentialdiagnostisch an Schlafstörungen im Allgemeinen und an *Restless-Legs-Syndrom (RLS)* im Besonderen denken sollte [34–36]. Explorierende Anamnese, strukturierte klinische Beobachtungen sowie eventuell die Auswertung von Videos tragen dazu bei, die Diagnose auch bei Kindern mit entwicklungsneurologischen Auffälligkeiten beschreibend zu stellen [34, 37, 38]. Da sich die pädiatrische Schlafmedizin an der Schnittstelle zwischen Vorsorgemedizin (Public Health), Versorgungsmedizin (im niedergelassenen Bereich) und klinischen Spezialdisziplinen (z. B. Entwicklungsneurologie/-psychologie, Kinder- und Jugendpsychiatrie) befindet, wird transdisziplinäres Denken zur Notwendigkeit, um Krankheitsbilder ganzheitlich und fachübergreifend erkennen zu können. Eine besondere Bedeutung kommt dabei den niedergelassenen KollegInnen zu, da die Klärung der Ursachen der Tagesmüdigkeit [39], die Interpretation des Blutbildes und das Erkennen des Eisenmangels mit Eisensubstitution als Therapie der ersten Wahl bei RLS [34, 36] – aber auch bei ADHS [32, 40] – schon im niedergelassenen Bereich erfolgen kann. Diese Gesichtspunkte werden in der AWMF-Neufassung der D‑A-CH-Leitlinie zum Restless-Legs-Syndrom angesprochen, fehlen aber noch in der ADHS-Leitlinie der AWMF. In aktuellen Multicenterstudien in Brandenburg und Sachsen wurden populationsbasierte Prävalenzdaten erhoben, in die 2183 bzw. 866 Probanden eingeschlossen wurden [41, 42]. Für eine erste Einschätzung steht ein validierter deutschsprachiger Fragebogen „Bewegungen im Schlaf bei Kindern“ (2019) zur Verfügung [43].

Manchmal steckt hinter Tagesmüdigkeit und Einschlafneigung bereits im Kindes- und Jugendalter eine *Narkolepsie*. Die neuen europäischen Richtlinien, welche auch die Behandlung von Kindern und Jugendlichen mit einbeziehen, bieten einen Überblick über aktuelle Behandlungsmöglichkeiten [44].

Die *COVID-19-Pandemie* war mit einer Zunahme von anamnestischen Hinweisen auf Schlafstörungen bei Kindern und Jugendlichen von 39 % auf 44 % vor bzw. während der Pandemie assoziiert [45]. Zum Thema Long Covid mit Erschöpfung und Müdigkeit in Abgrenzung zu Schlafstörungen im Kindes- und Jugendalter sind aktuelle Empfehlungen erschienen.

Die Einführung und Zulassung von *gentherapeutischen Medikamenten für Kinder mit spinaler Muskelatrophie bzw. mit Mukoviszidose* stellt einen epochalen Durchbruch in der Therapie dieser schweren Erkrankungen dar, die bisher mit einer deutlich begrenzten Lebenserwartung und u. a. auch mit der Aussicht auf außerklinische Beatmung verbunden sind. Zahlreiche Responder sprechen dafür, dass die Schlafmedizin und die Beatmungsmedizin zumindest bei einem Teil dieser Patienten entweder gar nicht mehr oder erst zu einem deutlich späteren Zeitpunkt einbezogen werden müssen [46].

Die Arbeitsgruppe Pädiatrie der DGSM hat 2018 eine Stellungnahme zum Einsatz von *Melatonin bei Kindern und Jugendlichen mit Schlafstörungen* herausgegeben. Auf der Grundlage der bis zu diesem Zeitpunkt publizierten randomisiert placebokontrollierten Studien zu dieser Altersgruppe konnten vier definierte Diagnosen bzw. Diagnosegruppen identifiziert werden, bei denen Melatonin in Bezug auf Einschlafstörungen wirksam sein kann: Delayed Sleep Phase Disorder (DSPD), ADHS, Autismus-Spektrum-Störungen sowie bei Kindern mit neurologischen Entwicklungsstörungen. Zu einigen seltenen Erkrankungen liegen Fallberichte bzw. Fallserien vor, die darauf hinweisen, dass Melatonin auch bei diesen Erkrankungen im Falle von Einschlafstörungen oder der Umkehr chronobiologischer Rhythmen wirksam sein kann (Smith-Magenis-Syndrom, Angelman-Syndrom, Rett-Syndrom und tuberöse Sklerose) [47]. Inzwischen gibt es auch Hinweise, über welche Melatoninrezeptoren und in welchen Hirnarealen Melatonin in Bezug auf die Schlafinduktion zu wirken scheint [48].

*Albträume, Insomnien und posttraumatische Belastungsstörungen* sind u. a. auch im Zusammenhang mit den Migrationswellen der letzten Jahre untersucht worden. Das Know-how zu diesem Thema, das bisher Kindern und Jugendlichen aus Afghanistan und anderen Ländern zugutegekommen ist, wird aktuell sicherlich auch ukrainischen Kindern und Jugendlichen helfen können, ihre ungewollten Erlebnisse und Erfahrungen verarbeiten zu können [49, 50]. Empfehlungen für mit ihren Kindern geflüchtete Eltern bei Schlafstörungen durch Kriegs- und Fluchterlebnisse im Kindesalter sollen erste Hilfestellungen bieten [51].

Die Bedeutung der freudschen *Traumforschung *ist in Bezug auf Kinder auf der Grundlage der kritischen Analyse seiner Texte deutlich infrage gestellt worden, da Freud für diese Altersgruppe nur eine minimale und unzureichend dokumentierte empirische Basis verwendet hat [5]. Der erste Bericht über einen Traum – und auch der erste Friedensvertrag der Menschheit – ist auf der Geierstele aus Mesopotamien in Keilschrift festgehalten worden [5]. Friedrich der Große hat zahlreiche Traumberichte ausführlich notiert oder seinem Leibarzt berichtet, in denen er Kindheitserlebnisse und traumatische Erfahrungen verarbeitet hat [5]. Carl Gustav Carus führte den Begriff des Unterbewussten schon einige Jahrzehnte vor Sigmund Freud ein [5]. Kritische Medizingeschichte weist darauf hin, dass Zitate auch Übersetzungsfehler aus alten Schriften enthalten können und dass alte Texte, Bilder, Plastiken und weitere Objekte nicht nur zitiert werden sollten, sondern dass deren Inhalt erst bei der Berücksichtigung kontextbezogener Variablen erschlossen werden kann [4, 52, 53] Osman Ipsiroglu und Angelika Schlarb plädieren in gleicher Weise dafür, Kinder sowie deren Eltern und Peers immer im Kontext ihrer Umgebungsbedingungen und Prägungen zu verstehen [38, 54–56].

## Perspektiven

Nachdem seitens der Arbeitsgruppe Pädiatrie der DGSM konkrete Empfehlungen für die schlafmedizinische Diagnostik bei Kindern erarbeitet wurden und für Kinder geltende Referenzwerte polysomnographischer Untersuchungen in multizentrischen Studien zusammengestellt wurden, liegt der Fokus innerhalb der DGSM darauf, den Stellenwert der Kinderschlafmedizin in der Pädiatrie weiterhin zu erhöhen. Positiv dazu beitragen können Lehrbuchbeiträge [57–60] und das 2020 erschienene Praxishandbuch Kinderschlaf [61], das den in Klinik und Praxis tätigen ÄrztInnen, aber auch kinderpsychologisch arbeitenden KollegInnen einen niederschwelligen Einstieg in die Kinderschlafmedizin ermöglicht. Die Anzahl der Kinderschlaflabore in Deutschland wird nach wie vor auch nicht annähernd dem Bedarf gerecht. Das führt dazu, dass Schlafstörungen bei Kindern unterdiagnostiziert und -behandelt werden. Kinderschlafmedizin spielt im Medizinstudium wie auch in der pädiatrischen Weiterbildung so gut wie keine Rolle. Das Thema wird weiterhin von vielen Entscheidungsträgern innerhalb der Kinder- und Jugendmedizin zu wenig beachtet. Die Arbeitsgruppe Pädiatrie der DGSM versucht dem entgegenzuwirken – durch regelmäßige Symposien auf pädiatrischen Kongressen und Publikationen, wie unter der Rubrik „Klug entscheiden“ im Deutschen Ärzteblatt [22]. Die Arbeitsgruppe sieht es aber auch als eine ihrer Aufgaben an, Erziehungsberechtigten Informationen zum Kinderschlaf zur Verfügung zu stellen (z. B. Empfehlungen zum sicheren Babyschlaf oder zum Mittagsschlaf im Kindergarten [62, 63]). Dieser Weg wird künftig noch nachhaltiger zu beschreiten sein.
